# Preoperative Upper Endoscopy and Surgical Specimen Findings in Bariatric Surgery Patients

**DOI:** 10.1007/s11695-025-07958-7

**Published:** 2025-06-05

**Authors:** Baila Elkin, Joseph El-Dahdah, Qijun Yang, Yueqi Wu, John McMichael, Michelle Kang Kim, Ricard Corcelles Codina, Carlos Roberto Simons Linares, Carol Rouphael

**Affiliations:** 1 Department of Internal Medicine, Cleveland Clinic, Cleveland, OH USA; 2 Department of Quantitative Health Sciences, Cleveland Clinic, Cleveland, OH, USA; 3 Department of General Surgery, Cleveland Clinic, Cleveland, OH, USA; 4 Department of Gastroenterology, Hepatology and Nutrition, Digestive Disease Institute, Cleveland Clinic, Cleveland, OH, USA; 5 Bariatric and Metabolic Institute, Department of General Surgery, Cleveland Clinic, Cleveland, OH, USA

**Keywords:** Obesity, Endoscopy, Metabolic bariatric surgery, Clinically significant findings, Missed lesions

## Abstract

**Background:**

The role of routine esophagogastroduodenoscopy (EGD) before metabolic bariatric surgery (MBS) remains unclear. We examined which patients were more likely to undergo preoperative EGD with biopsies, assessed the prevalence of clinically significant gastric pathologies on surgical specimens that may have been missed preoperatively, and analyzed patient factors associated with those findings.

**Methods:**

Patients ≥ 18 years old with Roux-en-Y gastric bypass (RYGB) or sleeve gastrectomy (SG) between 2018 and 2022 were included. Demographic, clinical, endoscopic, and pathology (from EGD and surgical specimens) characteristics were collected. Descriptive statistics were used. Uni- and multivariable Cox regression analyses assessed factors associated with clinically significant endoscopic and pathology findings.

**Results:**

Of 3718 patients (38.8% RYGB, 61.2% SG), 80% were female, 69.9% White, and 12.6% Hispanic. Median age at surgery was 45.7 years; median body mass index was 44.5 kg/m^2^. Preoperative EGD was performed in 36.9% of patients. Surgical specimens were available for 2349 patients (2273 SG and 76 RYGB), among whom 135 had clinically significant pathology. 63.7% of these patients had no preoperative EGD, 15.6% had preoperative EGD without biopsies, and 20.7% preoperative EGD with biopsies. Older age and non-White, non-Black race were associated with clinically significant findings on EGD or pathology.

**Conclusions:**

Several clinically relevant gastric pathologies could have been detected preoperatively via EGD with biopsies, yet about two-thirds did not undergo this evaluation. Older age and non-White, non-Black race were associated with these findings. Further research is needed to assess predictive factors of significant findings and cost-effectiveness of routine versus selective pre-operative EGD.

## Introduction

Metabolic-bariatric surgery (MBS) is an effective treatment option for patients with obesity, with sleeve gastrectomy (SG) and Roux-en-Y gastric bypass (RYGB) being the most commonly performed surgeries [1]. The role of esophagogastroduodenoscopy (EGD) prior to MBS remains controversial, particularly among asymptomatic patients. While preoperative EGD can help identify endoscopic or pathology findings that may alter surgical planning, it may lead to false positives and an overdiagnosis of pathologies in asymptomatic patients, resulting in unnecessary changes in surgical plans [2]. On the other hand, significant findings on preoperative EGD have been found in up to 25% of asymptomatic patients with a change in surgical plan in around 16% of them [3].

EGD findings that may impact surgical planning include endoscopic abnormalities like large hiatal hernias or reflux esophagitis, which are relative contraindications to SG [4, 5], and pathology findings such as pre-malignant or malignant lesions [6]. Atrophic gastritis and gastric intestinal metaplasia (GIM) may be relevant to surgical planning as RYGB and one-anastomosis gastric bypass surgery (OAGBS) leave a remnant stomach in-situ which is difficult to access endoscopically, making surveillance of pre-malignant gastric lesions difficult [7–10]. SG may hence be preferred over RYGB and OAGBS in such patients [7, 8]. In addition, gastric cancer in the remnant stomach has been described with OAGBS [9, 10], with concerns over whether biliary reflux after OAGBS predisposes to cancer [11, 12]. EGD can also identify *Helicobacter pylori* (*H. pylori*) which may result in surgical delays [6, 13, 14].

To date, none of the US societies recommend universal EGD before MBS, but support an individualized approach [5, 15, 16]. Multiple studies have examined the role of preoperative EGD [2, 6, 7, 13, 14, 17–26]; however, those evaluating surgical specimens rarely correlate these findings with preoperative EGD. At our center, there is no standard protocol for EGD before MBS, particularly in asymptomatic patients, leaving the decision to the surgeon’s discretion.

In this study, we explore the role of preoperative EGD prior to MBS by assessing which patients are more likely to undergo preoperative EGD with or without gastric biopsies, describing EGD findings, and evaluating the rate of gastric pathologies on surgical specimens from patients with no preoperative EGD. We also assess patient factors associated with clinically significant findings on either EGD or pathology.

## Methods

### Study Population and Definitions

This retrospective cohort study approved by our center’s institutional review board included patients ≥ 18 years old who underwent RYGB or SG between January 2018 and January 2022 and excluded those with hereditary cancer syndromes. Preoperative EGD was defined as EGD within one year of surgery date. Gastric surgical specimens were defined as specimens excised at the time of MBS. Clinically significant endoscopic findings were defined as findings that might alter or delay surgery and included EGD findings of esophagitis, esophageal ulcer, Barrett’s esophagus, stomach ulcer, stomach nodules, stomach atrophy, as well as pathology findings of *H. pylori*, atrophy, intestinal metaplasia, dysplasia, adenocarcinoma, mucosa-associated lymphoid tissue (MALT) lymphoma, and gastrointestinal stromal tumors (GIST).

### Data Source and Variables

Structured query language and natural language processing were used to identify patients who underwent RYGB or SG during the study period and collect patient demographics (date of birth, race, ethnicity, sex), clinical characteristics (body mass index (BMI) at time of preoperative EGD, smoking, alcohol use), preoperative EGD indication, esophageal and gastric endoscopic findings, and gastric pathology findings from both preoperative endoscopic and surgical specimens. Family history of gastric cancer was manually determined via chart review.

### Statistical Analysis

Data are described as median and interquartile range (IQR) for non-normally distributed continuous variables, and frequency (percentage) for categorical variables. Pearson’s Chi-square and Fisher’s exact tests compared categorical variables, and the Wilcoxon rank sum test compared continuous variables. Univariable and multivariable Cox regression analyses assessed patient factors associated with clinically significant findings on endoscopy or pathology. Analyses were performed using R software, and a significance level of 0.05 was assumed for all tests.

## Results

### Demographics and Clinical Characteristics

**Fig. 1:**
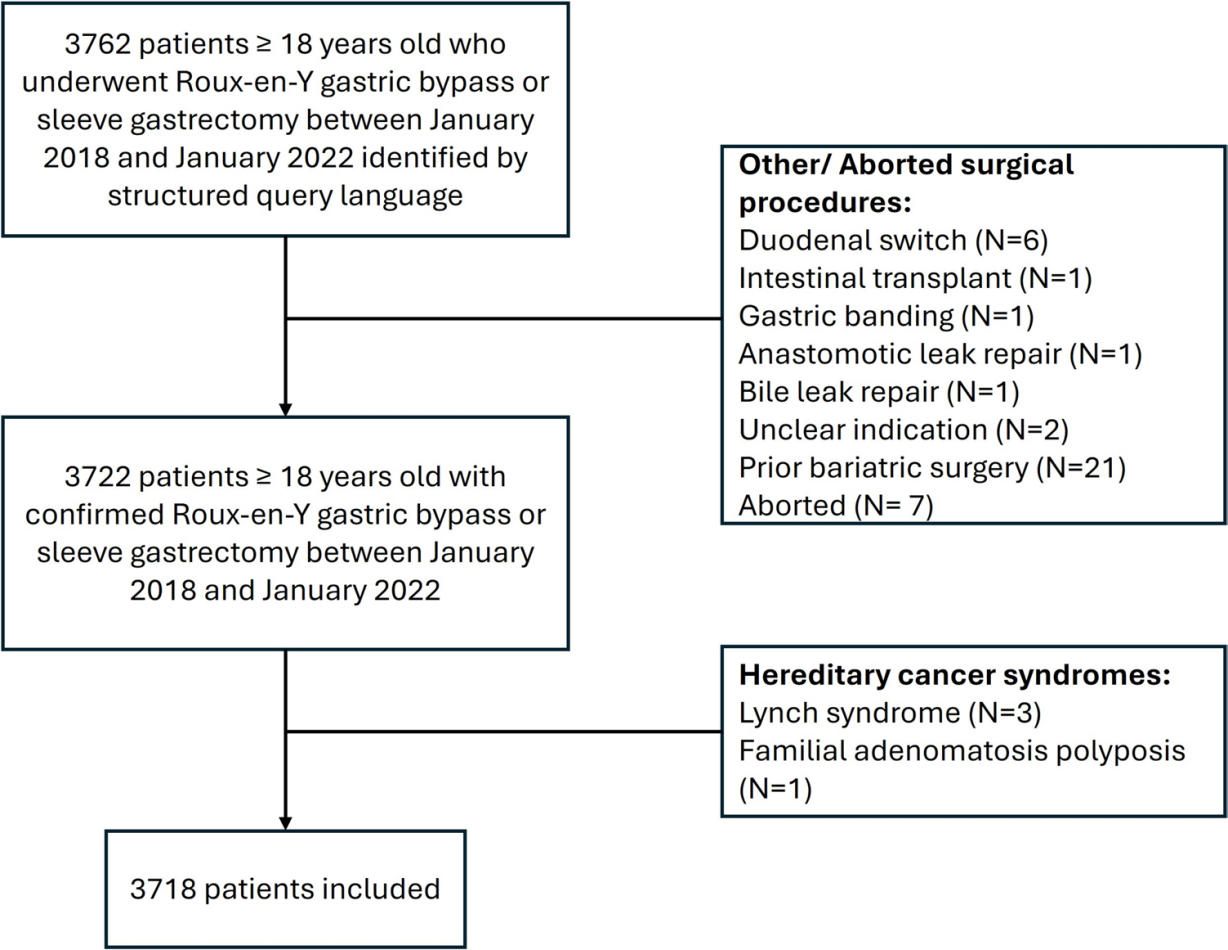
Flow diagram of included and excluded patients

A total of 3718 patients were included (Fig. 1). 38.8% underwent RYGB and 61.2% SG. Overall median age at the time of surgery was 45.7 years (IQR: 36.5; 54.7), and those undergoing RYGB were younger [44.9 years (IQR: 36.7; 53.5) vs. 46.2 (IQR: 36.4; 55.6) for SG patients (*p* = 0.015)]. Most patients were female (80%) and White (69.9%). Hispanic patients were more likely to undergo SG compared to RYGB surgery (15.8% vs. 7.7% respectively, *p* < 0.001). Median BMI at surgery was 44.5 kg/m^2^ (IQR 40.4; 49.6), with no significant difference in RYGB patients compared to SG patients (Table 1).

**Table 1:** Demographic and clinical characteristics of patients undergoing Roux-en-Y gastric bypass vs. sleeve gastrectomy

	All (*N* = 3718)	Roux-en-Y gastric bypass (*N* = 1443)	Sleeve gastrectomy (*N* = 2275)	*p* value
Age at surgery (years)	45.7 [36.5;54.7]	44.9 [36.7;53.5]	46.2 [36.4;55.6]	**0.015**
Sex				**0.004**
Male	742 (20.0%)	253 (17.5%)	489 (21.5%)	
Female	2976 (80.0%)	1190 (82.5%)	1786 (78.5%)	
Ethnicity (*N* = 3661)				**< 0.001**
Not Hispanic	3198 (87.4%)	1306 (92.3%)	1892 (84.2%)	
Hispanic	463 (12.6%)	109 (7.70%)	354 (15.8%)	
Race (*N* = 3628)				**< 0.001**
Black	877 (24.2%)	245 (17.4%)	632 (28.5%)	
White	2535 (69.9%)	1083 (76.9%)	1452 (65.4%)	
Other	216 (5.95%)	80 (5.68%)	136 (6.13%)	
Body mass index (kg/m^2^) at surgery	44.5 [40.4;49.6]	44.9 [40.6;49.4]	44.3 [40.3;49.8]	0.558
Tobacco use (*N* = 3716)				0.077
No	2337 (62.9%)	881 (61.1%)	1456 (64.0%)	
Yes	1379 (37.1%)	561 (38.9%)	818 (36.0%)	
Alcohol use (*N* = 3620)				0.465
No	1229 (34.0%)	465 (33.2%)	764 (34.4%)	
Yes	2391 (66.0%)	936 (66.8%)	1455 (65.6%)	
Family history of gastric cancer				0.442
No	3571 (96.0%)	1381 (95.7%)	2190 (96.3%)	
Yes	147 (3.95%)	62 (4.30%)	85 (3.74%)	

### Preoperative EGD

Overall, 36.9% (*N* = 1371) of patients undergoing MBS had preoperative EGD. Indication was listed for 1264 of these patients and was “preoperative” for more than half (56.6%) (*N* = 716), GERD/Barrett’s for 31.8% (*N* = 402), with 11.6% (*N* = 146) having other indications (i.e., gastrointestinal bleed, nausea/vomiting, dysphagia, cirrhosis). 63.5% (*N* = 870) of patients with preoperative EGD had gastric biopsies taken. Compared to those with no preoperative EGD, patients undergoing preoperative EGD were more likely to be older [median age 47.1 years vs. 44.7 years, *p* < 0.001], Hispanic (21.6% vs. 7.4%, *p* < 0.001), Black (25.0% vs. 23.7%, *p* < 0.001), have a lower BMI at surgery (43.1 kg/m^2^ vs. 45.4 kg/m^2^, *p* < 0.001), and undergo SG (70.4% vs. 55.8%, *p* < 0.001) (Table 2).

**Table 2:** Demographic and clinical characteristics of patients with preoperative EGD vs. none

	All (*N* = 3718)	No preoperative EGD (*N* = 2347)	Preoperative EGD (*N* = 1371)	*p* value
Age at surgery (years)	45.7 [36.5;54.7]	44.7 [35.9;53.8]	47.1 [38.0;56.0]	** < 0.001**
Sex				0.853
Male	742 (20.0%)	471 (20.1%)	271 (19.8%)	
Female	2976 (80.0%)	1876 (79.9%)	1100 (80.2%)	
Ethnicity (*N* = 3661)				**< 0.001**
Not Hispanic	3198 (87.4%)	2136 (92.6%)	1062 (78.4%)	
Hispanic	463 (12.6%)	170 (7.37%)	293 (21.6%)	
Race (*N* = 3628)				**< 0.001**
Black	877 (24.2%)	540 (23.7%)	337 (25.0%)	
White	2535 (69.9%)	1634 (71.6%)	901 (66.9%)	
Other	216 (5.95%)	107 (4.69%)	109 (8.09%)	
Body mass index (kg/m^2^) at surgery	44.5 [40.4;49.6]	45.4 [41.1;50.9]	43.1 [39.6;47.4]	**< 0.001**
Tobacco use (*N* = 3716)				**0.001**
No	2337 (62.9%)	1429 (60.9%)	908 (66.3%)	
Yes	1379 (37.1%)	917 (39.1%)	462 (33.7%)	
Alcohol use (*N* = 3620)				0.624
No	1229 (34.0%)	783 (34.3%)	446 (33.4%)	
Yes	2391 (66.0%)	1502 (65.7%)	889 (66.6%)	
Family history of gastric cancer				0.148
No	3571 (96.0%)	2263 (96.4%)	1308 (95.4%)	
Yes	147 (3.95%)	84 (3.58%)	63 (4.60%)	
Procedure name				**< 0.001**
Roux-en-Y	1443 (38.8%)	1037 (44.2%)	406 (29.6%)	
Sleeve gastrectomy	2275 (61.2%)	1310 (55.8%)	965 (70.4%)	

*EGD* Esophagogastroduodenoscopy

### Preoperative EGD and Pathology Findings

Among the 1371 patients who underwent preoperative EGD, 42.5% of those who had RYGB had “preoperative” as the indication for EGD compared to 62.6% of those who underwent SG (*p* < 0.001). RYGB patients were more likely to have esophagitis (22.2% vs. 8.08%, *p* < 0.001) and less likely to have Barrett’s esophagus (5.91% vs. 7.25%, *p* < 0.001) on preoperative EGD compared to SG patients (Table 3). Overall, 8.16% (*N* = 71) had *H. pylori* on preoperative EGD, 2.64% (*N* = 23) had GIM, 1 patient had gastric dysplasia, and none had gastric atrophy, MALT lymphoma, or gastric adenocarcinoma.

**Table 3:** Endoscopic characteristics of patients undergoing Roux-en-Y gastric bypass vs. sleeve gastrectomy

	All (*N* = 1371)	Roux-en-Y gastric bypass (*N* = 406)	Sleeve gastrectomy (*N* = 965)	*p* value
Indication (*N* = 1264)				** < 0.001**
GERD/Barrett’s	402 (31.8%)	153 (41.1%)	249 (7.9%)	
Preoperative	716 (56.6%)	158 (42.5%)	558 (62.6%)	
Other	146 (11.6%)	61 (16.4%)	85 (9.53%)	
Endoscopic finding				
Esophagitis	168 (12.3%)	90 (22.2%)	78 (8.08%)	** < 0.001**
Esophageal ulcer	4 (0.29%)	1 (0.25%)	3 (0.31%)	1.000
Esophageal nodule	7 (0.51%)	1 (0.25%)	6 (0.62%)	0.681
Barrett’s esophagus	37 (2.07%)	24 (5.91%)	70 (7.25%)	** < 0.001**
Stomach erythema	94 (6.86%)	24 (5.91%)	70 (7.25%)	0.435
Stomach ulcer	16 (1.17%)	6 (1.48%)	10 (1.04%)	0.582
Stomach congestion	1 (0.07%)	0 (0.00%)	1 (0.10%)	1.000
Stomach erosion	56 (4.08%)	17 (4.19%)	39 (4.04%)	1.000
Stomach nodule	15 (1.09%)	3 (0.74%)	12 (1.24%)	0.573
Stomach atrophy	15 (1.09%)	3 (0.74%)	12 (1.24%)	0.701

*GERD* Gastroesophageal reflux disease

### Findings on Gastric Surgical Specimens and Follow-Up

A total of 2349 patients had gastric surgical specimens submitted at the time of surgery (2273 from SG and 76 RYGB). Of those, there were 135 unique patients with clinically significant gastric pathology on surgical specimens including GIM in 24 patients, *H. pylori* in 88, gastric mucosal atrophy in eight, MALT lymphoma in two and GISTs in 26 patients. All 24 patients with GIM underwent SG, ten had preoperative EGD of which three had gastric biopsies, with one showing GIM, and three patients had follow-up with recommendations for surveillance or mapping EGD. Of the 88 patients with *H. pylori*, 87 underwent SG and one RYGB, 31 had preoperative EGD, of whom 17 had gastric biopsies, ten of which showed *H. pylori*, and 57 patients had post-operative follow-up with 52 receiving treatment. Of the eight patients with gastric atrophy, all underwent SG, four had preoperative EGD, two with gastric biopsies, neither showing atrophy, and four had follow-up with surveillance or mapping EGD recommended. Two patients had MALT lymphoma on their surgical specimens, and both underwent SG with neither undergoing preoperative EGD. Both patients had follow-up referrals to oncology, resulting in EGD confirming eradication of malignancy in one, and annual CT scan for surveillance in the other. Of the 26 patients with GIST on surgical pathology, 18 underwent SG, seven had preoperative EGD, six had any gastric biopsies, and one had a non-diagnostic biopsy of a submucosal lesion. Eighteen had follow-up for GIST, the majority with referral to medical oncology. Of the eight without follow-up, seven had negative margins noted on pathology, and one had benign pathology findings of leiomyoma.

### Factors Associated with Clinically Significant Endoscopic and Pathology Findings

There were 2733 unique patients who underwent either preoperative EGD (with or without biopsies) or had surgical specimens available. Of those, 329 had a clinically significant finding endoscopically or on pathology specimens (endoscopic or surgical). Older patients were more likely to have findings. Additional comparative characteristics are presented in Table 4. On multivariable analysis, older age [odds ratio (OR) 1.01 (95% confidence interval (CI) 1.00, 1.02], non-White, non-Black race [OR 1.58 (95% CI 1.02, 2.40)], but not BMI [OR 0.98, (95% CI 0.97, 1.00)] were associated with clinically significant endoscopic or pathology findings (Table 5).

**Table 4:** Demographic and clinical characteristics of patients with any clinically significant endoscopic or pathology findings versus none

	All (*N* = 2733)	Clinically significant findings absent (*N* = 2404)	Clinically significant findings present (*N* = 329)	*p* value
Age at surgery (years)	46.4 [36.6;55.6]	46.1 [36.4; 55.1]	49.2 [38.9; 58.8]	< 0.001
Gender				0.626
Male	574 (21.0%)	501 (20.8%)	73 (22.2%)	
Female	2158 (79.0%)	1902 (79.2%)	256 (77.8%)	
Ethnicity				0.064
Not Hispanic	2289 (84.8%)	2025 (85.3%)	264 (81.2%)	
Hispanic	409 (15.2%)	348 (14.7%)	61 (18.8%)	
Race				0.053
Black	706 (26.5%)	637 (27.2%)	69 (21.4%)	
White	1793 (67.2%)	1565 (66.7%)	228 (70.6%)	
Other	170 (6.37%)	144 (6.14%)	26 (8.05%)	
BMI at surgery	44.2 [40.1; 49.5]	44.3 [40.2; 49.9]	43.0 [39.7;47.7]	< 0.001
Tobacco use				1.000
No	1738 (63.6%)	1529 (63.6%)	209 (63.5%)	
Yes	994 (36.4%)	874 (36.4%)	120 (36.5%)	
Alcohol use				0.883
No	925 (34.7%)	815 (34.8%)	110 (34.2%)	
Yes	1742 (65.3%)	1530 (65.2%)	212 (65.8%)	
Family history of gastric cancer				0.499
No	2623 (96.0%)	2310 (96.1%)	313 (95.1%)	
Yes	110 (4.02%)	94 (3.91%)	16 (4.86%)	
Surgery type				< 0.001
Roux-en-Y	459 (16.8%)	327 (13.6%)	132 (40.1%)	
Sleeve gastrectomy	2274 (83.2%)	2077 (86.4%)	197 (59.9%)

**Table 5:** Uni- and multivariable analyses for clinically significant findings

	Univariable analysis			Multivariable analysis		
Characteristic	OR	95% CI	*p* value	OR	95% CI	*p* value
Age at surgery (years)	1.01	1.00, 1.02	0.002	1.01	1.00, 1.02	**0.003**
Gender						
Male	—	—		—	—	
Female	0.99	0.77, 1.28	0.943	1.00	0.77, 1.31	> 0.99
Ethnicity						
Not Hispanic	—	—		—	—	
Hispanic	1.16	0.87, 1.53	0.303	1.11	0.80, 1.51	0.54
Race						
White	—	—		—	—	
Black	1.14	0.89, 1.44	0.292	1.20	0.93, 1.55	0.15
Other	1.51	1.01, 2.22	0.039	1.58	1.02, 2.40	**0.036**
BMI at surgery	0.98	0.96, 0.99	0.003	0.98	0.97, 1.00	**0.024**
Tobacco use						
No	—	—		—	—	
Yes	1.00	0.80, 1.24	0.985	1.01	0.80, 1.27	0.92
Alcohol use						
No	—	—		—	—	
Yes	0.88	0.71, 1.09	0.242	0.89	0.71, 1.12	0.32
Family history of gastric cancer						
No	—	—		—	—	
Yes	1.15	0.68, 1.87	0.580	1.11	0.65, 1.81	0.69

*OR* odds ratio, *CI* confidence interval

## Discussion

There is currently no consensus on the role of preoperative EGD in MBS, resulting in practice heterogeneity. Reported rates of EGD findings that alter surgical planning range from 3.9% [5] to 23.8% [13], contributing to ongoing debate between advocates for routine preoperative EGD [4, 7, 13, 18, 20, 24], and those supporting an individualized approach [5, 6, 15, 16, 21]. In our study, several clinically significant gastric pathologies identified on surgical specimens could have been detected preoperatively. However, only 20.7% of those patients had preoperative EGD with gastric biopsies. This suggests that routine preoperative EGD with biopsies may help identify relevant gastric pathologies before MBS. We found that older age and non-White, non-Black race were associated with such findings.

At our institution, 36.9% of patients had preoperative EGD with more than half done for a routine “preoperative” indication. Rates of preoperative EGD have been shown to range from 2.2% to 97% [7], highlighting practice heterogeneity and a need for a standardized approach. Studies advocating for an individualized approach primarily support a symptom-driven strategy [5, 15, 16, 23], while others suggest that overall risk and surgery type should also be considered in the decision-making process [21]. We found older and Hispanic patients were more likely to undergo preoperative EGD. While both older age and Hispanic ethnicity are recognized risk factors for gastric cancer [27], it is unclear whether this awareness drives the decision for preoperative EGD at our institution. Interestingly, older age and non-White, non-Black race, but not Hispanic ethnicity, were associated with the presence of clinically significant findings, suggesting that those patients may benefit the most if an individual approach to preoperative endoscopy is used. However, further studies are needed to better define clinical characteristics of patients most likely to benefit from preoperative EGD. Surgery type is an important factor in the decision for preoperative EGD, with some studies advocating for preoperative EGD prior to SG but not RYGB [19]. In consonance with this, we found that patients undergoing SG had higher rates of preoperative EGDs at our institution (42.4% vs. 28.1% in RYGB patients).

The overall prevalence of GIM in our bariatric population with gastric pathology was 1.79%, consistent with other studies [6, 7]. In our study, among patients with GIM found on surgical pathology, only 41.7% had undergone preoperative EGD, and only 12.5% had undergone preoperative gastric biopsy. These findings highlight opportunities for improving detection rates prior to surgery. GIM findings on preoperative EGD can be clinically relevant if extensive in the stomach and may impact surgical planning, since a RYGB leaves a remnant stomach that is not easily accessible for GIM surveillance, leading some surgeons to favor SG in such cases.

We found 8% of patients with preoperative EGD and gastric biopsies had *H. pylori*. The prevalence of *H. pylori* detected on preoperative EGD before MBS varies widely in the literature, with rates ranging between 3.7% [24] and 46.67% [18]. This is likely partly due to the regional variations in *H. pylori* prevalence. Some guidelines, such as the American Society for Metabolic and Bariatric Surgery, take this variation into account, with a Grade C recommendation for *H. pylori* screening preoperatively in high-prevalence areas, but do not specify EGD as the preferred screening modality [16]. Similarly to our GIM findings, among patients with *H. pylori* found on surgical pathology, 35.2% had undergone preoperative EGD, and 19.3% had preoperative gastric biopsy, again highlighting opportunities for detecting *H. pylori* prior to surgery. Findings of *H. pylori* are clinically relevant, and may delay surgical plans [5]. We found two cases of MALT lymphoma in resected gastric specimens of patients who did not undergo preoperative EGD. While the rate is low at 0.085%, this underscores the importance of preoperative EGD in identifying clinically significant pathology. While most studies do not comment on the rates of MALT lymphoma specifically, malignancy in general is a rare finding [7, 21–24]. The most common tumor found incidentally during MBS is a GIST [28]. We found 26 patients with GIST in our cohort. The incidental diagnosis and management of GIST during MBS is primarily a surgical consideration. Surgical considerations of incidental GIST range from inclusion of a wedge resection, alteration of the surgical plan, to opting for resection with negative margins in favor of MBS when resection precludes the bariatric procedure [28]. Preoperative EGD can hence be useful in diagnosing submucosal lesions prior to surgery, which would assist with surgical planning. Our surgical gastric pathology findings were derived from SG patients. Since RYGB typically does not result in routine gastric pathology specimens, we are unable to directly assess the number of gastric pathologies in this population; however, we can extrapolate a comparable number based on the SG population.

While cost-effectiveness of preoperative EGD was beyond the scope of our study, it remains an important consideration. Some studies suggest that preoperative EGD may have a high-cost with a relatively low diagnostic yield [23]. Others point out that the cost for patients is variable depending on the specific practice model and insurance [2]. Resource availability is another critical factor, particularly in underserved settings where access to endoscopy may be limited. Further studies are needed to investigate cost-effectiveness and provide a risk–benefit assessment of routine preoperative EGD.

Our study has several limitations; it is a retrospective, single center study from a tertiary care facility, which may not fully represent the broader population undergoing MBS, potentially introducing selection bias. Additionally, because preoperative EGD is at the surgeon’s discretion at our center, there is potential for selection bias in which patients underwent EGD. Our study also has notable strengths, namely the large number of patients and the detailed manual chart review of patients found to have gastric pathology on their surgical specimens, allowing a granular analysis of missed opportunities for preoperative detection of gastric pathology. By correlating findings on surgical specimens with preoperative EGDs, our study adds a unique perspective.

In conclusion, we found several clinically relevant gastric pathologies on excised surgical specimens that could have been detected preoperatively through EGD or gastric biopsies. However, fewer than 25% of those patients had preoperative EGD with gastric biopsies, highlighting potential missed diagnostic opportunities before MBS. We found older age and non-White, non-Black race were associated with these findings, suggesting that certain patient characteristics may help guide decision-making for preoperative EGD. To determine if a universal or individualized approach is more appropriate, further research is needed to evaluate factors associated with clinically significant endoscopic and pathology findings and the cost-effectiveness of routine EGD prior to MBS.

## Data Availability

No datasets were generated or analysed during the current study.
